# An Epidemiologic Analysis of Associations between County-Level Per Capita Income, Unemployment Rate, and COVID-19 Vaccination Rates in the United States

**DOI:** 10.3390/ijerph19031755

**Published:** 2022-02-03

**Authors:** Yuqi Guo, Andrea R. Kaniuka, Jingjing Gao, Omar T. Sims

**Affiliations:** 1 School of Social Work, College of Health and Human Services, University of North Carolina at Charlotte, Charlotte, NC 28262, USA; 2 School of Data Science, University of North Carolina at Charlotte, Charlotte, NC 28262, USA; 3 Department of Public Health Sciences, College of Health and Human Services, University of North Carolina at Charlotte, Charlotte, NC 28262, USA; akaniuka@uncc.edu; 4 Public Policy Program, College of Arts and Sciences, University of North Carolina at Charlotte, Charlotte, NC 28262, USA; jgao9@uncc.edu; 5 Department of Social Work, College of Arts and Sciences, University of Alabama at Birmingham, Birmingham, AL 35222, USA; 6 Department of Health Behavior, School of Public Health, University of Alabama at Birmingham, Birmingham, AL 35222, USA; 7 Center for AIDS Research, School of Medicine, University of Alabama at Birmingham, Birmingham, AL 35222, USA; 8 African American Studies, College of Arts and Science, University of Alabama at Birmingham, Birmingham, AL 35222, USA

**Keywords:** COVID-19 vaccination rates, race/ethnicity, per capita income, unemployment rate, racial disparities

## Abstract

The purpose of this longitudinal study was to examine associations between per capita income, unemployment rates, and COVID-19 vaccination rates at the county-level across the United States (U.S.), as well as to identify the interaction effects between county-level per capita income, unemployment rates, and racial/ethnic composition on COVID-19 vaccination rates. All counties in the U.S. that reported COVID-19 vaccination rates from January 2021 to July 2021 were included in this longitudinal study (*n* = 2857). Pooled ordinary least squares (OLS) with fixed-effects were employed to longitudinally examine economic impacts on racial/ethnic disparities on county-level COVID-19 vaccination rates. County-level per capita income and county-level unemployment rates were both positively associated with county-level COVID-19 vaccination rates across the U.S. However, the associations were divergent in the context of race/ethnicity. Public health efforts to bolster COVID-19 vaccination rates are encouraged to consider economic factors that are associated with decreases in COVID-19 vaccination rates.

## 1. Introduction

In February 2020, the novel coronavirus (COVID-19) was declared a public health emergency in the United States (U.S.), and in March 2020 it was declared a pandemic by the World Health Organization (WHO) [1]. Soon after, states in the U.S. began implementing various community mitigation strategies (e.g., mandatory stay at home orders and business closures) to curb the spread of COVID-19. In total, 42 U.S. states and territories issued mandatory stay-at-home orders, covering 73% of U.S. counties [2]. Community mitigation strategies were effective in their aim of reducing close contact and movement outside of households [2], and consequently reduced the number of COVID-19 cases [3]; however, these public health strategies were associated with an array of negative economic impacts, including higher unemployment rates, decreased participation in the labor force, and reductions in income. For example, the most recent estimates indicate that the unemployment rate peaked in April 2020 (14.8%) during the pandemic, and the current unemployment rate remains higher than the pre-pandemic unemployment rate (5.4% vs. 3.5%) [4]. Since the start of the pandemic, over 100 million unemployment claims have been filed, with one in four workers accessing unemployment aid at some point during the pandemic [5]. Furthermore, approximately one in five U.S. adults reported a drop in income during the pandemic, resulting in difficulty covering various expenses (e.g., rent or mortgage payments, medical care, and food costs) [6].

Certain demographic groups in the U.S. have been disproportionately affected by the economic impacts of COVID-19. Socio-economic status is significantly associated with health status and socio-economic factors represent important risk factors for disparities in health status [7]. In the U.S., individuals who are Black, Indigenous, People of Color (BIPOC) are more likely to experience unemployment or a reduction in income during the pandemic [6,8]. This trend is likely due to the racial/ethnic composition of workers in the sectors hardest hit during the COVID-19 pandemic. For example, the leisure (e.g., travel industry) and hospitality (e.g., restaurant workers) sectors, industries in which BIPOC individuals are more likely to work, saw the largest increases in unemployment [9]. Further, compared to White individuals, BIPOC individuals, who are already more likely to work in lower paying jobs [6], were more likely to report reductions in income and to have difficulty paying their bills [9]. As well, BIPOC individuals are reporting slower job recovery than White individuals [10]. The economic impacts of COVID-19 further exacerbated wealth and income gaps between White and BIPOC Americans [11] and compounded issues of access to a paramount public health prevention strategy—COV ID-19 vaccination. 

In order to curb the spread of COVID-19, multiple COVID-19 vaccinations were rapidly developed and eventually emerged as the primary public health approach to combat the COVID-19 pandemic [12]. Three COVID-19 vaccinations were granted emergency use authorization: the Pfizer-BioNTech and Moderna COVID-19 vaccines (December 2020) and the Johnson & Johnson (J&J) COVID-19 vaccine (February 2021) [13]. In August 2021, the Pfizer-BioNTech vaccine became the first to receive full FDA approval [14]. All three COVID-19 vaccines are effective, with twice vaccinated individuals being five times less likely to acquire COVID-19 infection and ten times less likely to experience hospitalization and death compared to unvaccinated individuals [15]. Despite their effectiveness as a primary prevention strategy, rates of vaccination lag behind desired targets set by the federal government [16]. The administration of vaccines began in December 2020, and by 24 January 2022, approximately 63.4% of Americans (~210.5 million) have been twice vaccinated (i.e., one shot of J&J vaccine, two doses of Pfizer or Moderna vaccine) [17].

Vaccination rates among BIPOC persons are lagging compared to their non-Hispanic White counterparts, with non-Hispanic Black and Hispanic Americans being less likely than non-Hispanic Whites to be twice vaccinated against COVID-19 [18]. Disparities in vaccination rates may be due to issues of access (e.g., lack of accessible clinic, inability to take time off of work), as well as vaccine hesitancy potentially rooted in mistrust in the medical field due to historical and contemporary experiences of healthcare discrimination [19,20]. This is of particular concern given that BIPOC persons have a high frequency of several COVID-19 risk factors (e.g., diabetes, heart disease, and obesity) [21]. As well, BIPOC individuals are more likely to work in “essential” jobs (e.g., factories, health care), and thus are less likely to be able to telework, ultimately increasing their exposure to COVID-19 [22]. As a result, compared to White Americans, BIPOC Americans have higher rates of COVID-19 infection and death [23], highlighting the importance of COVID-19 vaccination for this population and underscoring the need to address factors contributing to inequities in vaccine distribution.

As such, the Centers for Disease Control and Prevention (CDC) has identified COVID-19 vaccine equity for BIPOC individuals as a top priority, highlighting income and wealth gaps and employment as barriers to vaccination [24]. Burgeoning evidence suggests that at the individual and county level, household income and employment impact vaccination rates [25,26,27]. Furthermore, the extant literature suggests that social vulnerability, which takes into account the racial/ethnic composition of an area, is associated with lower vaccination rates [28]. 

However, social vulnerability is an aggregate score of all three factors which fails to allow for an examination of how unemployment rates and income may impact racial/ethnic disparities on COVID-19 vaccination rates. As such, using longitudinal data from the U.S. Census Bureau and the CDC, this study conducted a longitudinal analysis across the U.S. at the county-level (1) to examine the relationship between county-level per capita income and county-level COVID-19 vaccination rates, (2) to examine the relationship between county-level unemployment rates and county-level COVID-19 vaccination rates, and (3) to identify interaction effects between county-level per capita income, county-level unemployment rates, and county-level racial/ethnic composition on county-level COVID-19 vaccination rates. 

## 2. Materials and Methods

### 2.1. Study Design

An analysis of publicly available, secondary data was conducted in the U.S. at the county-level. County-level socio-economic demographics and county-level vaccination rates were extracted from the U.S. Census Bureau [29] and the CDC’s COVID-19 vaccine tracker [29], respectively. All U.S. counties that reported COVID-19 vaccination rates from January 2021 to July 2021 were included in the sample (*n* = 2857). This time span included seven time points, namely the first day of the month spanning January 2021 to July 2021. In total, the present study analyzed 19,999 county-time waves.

### 2.2. Dependent Variable

The dependent variable was the county-level adult vaccination rate, defined as the percentage of twice vaccinated adults (age 18 or older) per county on the first day of each month (January 2021 to July 2021), as reported by the CDC’s COVID-19 vaccine tracker [30]. 

### 2.3. Independent Variables 

County-level unemployment rates were measured by the number of unemployed adults in each county divided by the number of adults in the labor force in each county, as indicated by U.S. Census Bureau data. Using U.S. Census Bureau data [29], county-level per capita income was calculated by dividing the county’s total income by its population. 

### 2.4. Moderating Variable

County-level racial/ethnic composition was measured by the percentage of BIPOC adults in each county as reported by the U.S. Census Bureau [28]. This percentage was then dichotomized into the top and bottom 5% of the distribution by county-level racial/ethnic composition. In this study, BIPOC refers to all people of color including but not limited to Black, Hispanic, and Asian individuals.

### 2.5. Covariates

Covariates included access to the COVID-19 vaccine (i.e., number of days the COVID-19 vaccine was available in each county), the number of nurse practitioners in each county—a proxy for healthcare availability at the county-level, gender (male or female), education (percentage of adults with a bachelor’s degree or higher), and the percentage of individuals who were older adults (≥65 years old).

### 2.6. Data Analysis

Measures of central tendency and frequency distributions were used to characterize the study sample. Pooled ordinary least squares (OLS) with fixed-effects were employed to longitudinally examine economic impacts on racial/ethnic disparities on county-level COVID-19 vaccination rates. Interaction effects between the percentage of BIPOC adults and economic factors (i.e., unemployment, per capita income) on county-level vaccination rates were analyzed using OLS models with fixed-effects.

## 3. Results

Table 1 contains descriptive statistics across 19,999 county-time-waves (2857 counties from January 2021 to July 2021). Across time-waves, the average county-level COVID-19 vaccination rate was 14.82% (SD = 15.22), the mean racial/ethnic composition of counties with BIPOC was 15.45% (SD = 0.16), and the average number of days that the COVID-19 vaccine was available to the general population was 25.29 (SD = 33.35). The average unemployment rate was 6.71% across time-waves (SD = 2.24), while the average per capita income was $25,000.92 (SD = $5921.20). 

### 3.1. Associations between County-Level Per Capita Income, County-Level Unemployment Rate and County-Level Vaccination Rates

Table 2 presents the results of the pooled OLS with fixed-effects. Aim 1 was to assess the relationship between county-level per capita income and county-level COVID-19 vaccination rates. Per capita income was positively associated with COVID-19 vaccination rates. For every $10,000 dollar increase in per capita income, county-level COVID-19 vaccination rates increased by 0.01%. Aim 2 was to assess the relationship between county-level unemployment and county-level COVID-19 vaccination rates. The unemployment rate was positively associated with COVID-19 vaccination rates. For every 1% increase in unemployment rate, county-level COVID-19 vaccination rates increased by 0.41%.

### 3.2. Interaction Effects 

**Figure 1:**
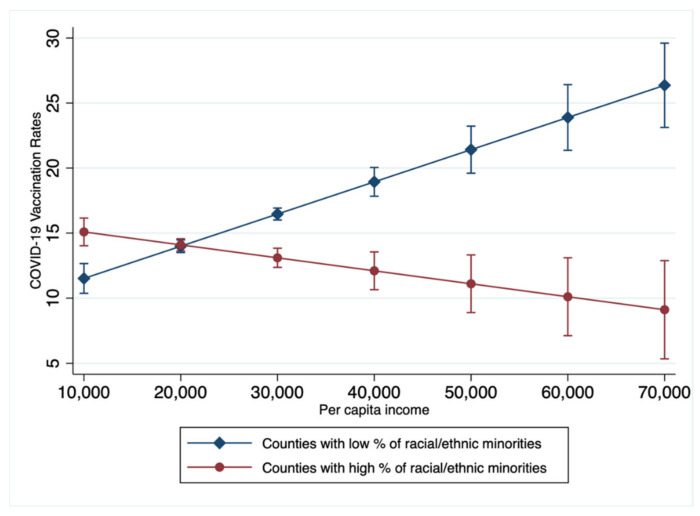
Interaction effects between County-level Per Capita Income, Racial/Ethnic Minorities, and COVID-19 Vaccination Rates. Counties with low and high percent of racial/ethnic minorities: the percentage of adults who are Black, Indigenous, People of Color adults was dichotomized into the top and bottom 5% of the distribution by county-level racial/ethnic composition.

**Figure 2:**
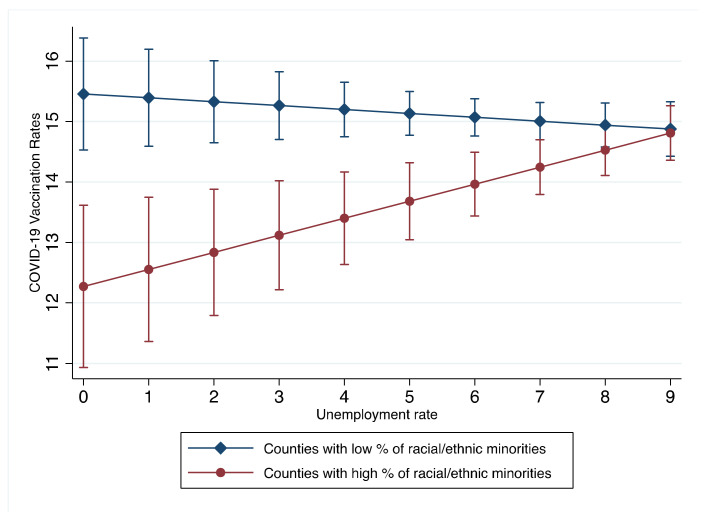
Interaction effects between County-level Unemployment Rate, Racial/Ethnic Minorities, and COVID-19 Vaccination Rates. Counties with low and high percent of racial/ethnic minorities: the percentage of adults who are Black, Indigenous, People of Color adults was dichotomized into the top and bottom 5% of the distribution by county-level racial/ethnic composition.

Aim 3 was to analyze interaction effects among county-level per capita income and unemployment rates with racial/ethnic composition (% of BIPOC adults) on county-level COVID-19 vaccination rates. Significant interaction effects were found between the unemployment rates and the percentage of racial minorities. A graph of the interaction effect is presented in Figure 1. In counties with greater racial/ethnic minority populations, increases in per capita income were associated with lower vaccination rates; however, in counties with lower racial/ethnic minority populations, increases in per capita income were associated with higher vaccination rates. Significant interaction effects were also found between the unemployment rate and the percentage of racial/ethnic minorities. A graph of the interaction effect is presented in Figure 2. In counties with greater racial/ethnic minority populations, increases in unemployment rates were related to higher COVID-19 vaccination rates; however, in counties with lower racial/ethnic minority populations, increases in unemployment rates were related to lower COVID-19 vaccination rates.

## 4. Discussion

This study longitudinally examined county-level relationships between county-level economic factors (i.e., per capita income and unemployment rate) and racial/ethnic composition and county-level COVID-19 vaccination rates in the U.S. Several notable findings emerged from the longitudinal analysis. First, county-level per capita income was positively associated with county-level COVID-19 vaccination rates across U.S counties, and similar findings have been found elsewhere at the county level [28]. Interestingly, we found that this trend (i.e., increases in per capita income being associated with increases in COVID-19 vaccination rates) was divergent in the context of interactive effects with race/ethnicity. We found that increases in per capita income were associated with decreases in COVID-19 vaccination rates in counties with higher proportions of BIPOC adults. It is plausible that race-based political ideology, unequal health care resource distribution, lack of culture-sensitive public health policies, medical distrust, and contemporary healthcare discrimination may contribute to this negative association between per capita income and COVID-19 vaccination rates in counties with higher proportions of BIPOC adults [19,20,31,32,33,34]. More studies are needed to explore reasons why COVID-19 vaccination rates in counties with higher proportions of BIPOC adults decrease with increasing per capita income. Despite state and national efforts to address racial inequalities in COVID-19 vaccination, without developing policy interventions that consider economic factors, lagging vaccination rates among BIPOCs will worsen. 

Second, county-level unemployment rates were positively associated with county-level COVID-19 vaccination rates. This finding is consistent with a prior study during the first 100 days of COVID-19 vaccination in the U.S., which found that higher state-level unemployment rates were associated with higher state-level vaccination rates [27]. However, we found that county-level proportions of BIPOC adults moderated the effects of county-level unemployment rates on county-level COVID-19 vaccination rates. Increases in unemployment rates were associated with increases in COVID-19 vaccination rates in counties with a higher proportion of BIPOC, but increases in unemployment rates were associated with decreases in COVID-19 vaccination rates in counties with lower proportions of BIPOC (i.e., predominantly non-Hispanic White). In general, unemployment is known to negatively impact vaccination rates for other viral infections (e.g., influenza), but findings from this study suggest that unemployment does not impact COVID-19 vaccination rates in a similar fashion between BIPOC and non-BIPOC individuals at the population-level [35,36]. Interestingly, since BIPOC individuals have faced higher risks of unemployment during the COVID-19 pandemic [9], they may be more motivated to vaccinate against COVID-19 in order to return to the workforce [37]. Equally important, unlike other types of vaccinations, the need for COVID-19 vaccination is not driven by the presence of a pressing pandemic, and the COVID-19 vaccination is widely available at no cost for those who are unemployed or without health insurance [38]. It is plausible that no-cost access to the COVID-19 vaccine for those unemployed and likely without health insurance in some way may provide a means for those with increased risk of unemployment, in particular BIPOC individuals, to secure employment, especially provided that many employers are starting to require COVID-19 vaccination. However, future studies are needed to explore and determine what situational or underlying mechanisms of the COVID-19 pandemic lead to increases in COVID-19 vaccination at the population-level among BIPOC individuals who are unemployed.

This study had notable limitations and strengths. Causality cannot be inferred given the study design and statistical approach. Vaccine incentive programs may bolster vaccination, and the current analysis did not include vaccine incentive programs in the analysis. Also, the study did not explore other social factors, such as index of deprivation and geographical (including but not limited to urban and rural) differences. Future studies may consider comparing COVID-19 vaccination differences based on geographics and socio-economic classifications. However, all counties in the U.S. were included in the study, which considerably increased the study’s generalizability. Unlike many studies, the study aimed to examine ways in which economic factors may contribute to disparities and impact outcomes in the context of race/ethnicity versus an examination of disparities and outcomes only based on race/ethnicity. 

## 5. Conclusions

Our findings indicate that county-level per capita income is negatively associated with county-level COVID-19 vaccination rates in counties with higher proportions of BIPOC individuals, while the county-level unemployment rate is negatively associated with county-level vaccination rates in counties with higher proportions of non-Hispanic White individuals. Taken together, it is critical to develop policy interventions to increase vaccination rates in racial/ethnic minority communities in order to stimulate economic recovery. Public health efforts to bolster COVID-19 vaccination rates are encouraged to consider and respond to economic factors that are associated with decreases in COVID-19 vaccination rates. Future research exploring factors underlying these disparate findings at the county-level across the U.S. in the context of race/ethnicity are needed.

## Figures and Tables

**Table 1:** Descriptive Statistics.

*n* = 19,992				
County-Level Variables	Mean	Std. Dev.	Min	Max
Panel time range			1 January 2021	7 January 2021
COVID-19 vaccination rate	14.82	15.22	0.00	99.90
Percentage of Black and Indigenous People of Color (BIPOC)	15.45	16.12	0.91	93.71
Females	49.99	2.12	26.84	56.87
Number of days of COVID-19 vaccine availability	25.29	33.35	0.00	107.00
Number of nurse practitioners	53.04	154.49	1.00	3938.00
Unemployment rate	6.71	2.24	1.70	22.50
Per capita income	25,000.92	5921.20	9688.43	66,518.36
Percent of adults with bachelor’s degree	21.82	9.55	5.40	78.50
Percentage of older adults population aged (≥65 years old)	19.42	4.58	4.83	57.59

**Table 2:** Ordinal Least Squared Analysis Examining Associations with County-level COVID-19 Vaccination Rates and Interaction Effects.

	County-Level COVID-19 Vaccination Rates		
County-Level Variables	Time-Fixed Effects	Unemployment Rate	Per Capita Income
Rates of racial minorities	−4.583 ***	−10.50 ***	14.96 ***
Number of nurse practitioners	0.00276 ***	0.00268 ***	0.00431 ***
Unemployment rate	0.413 ***	0.296 ***	0.421 ***
Per capita income	0.000132 ***	0.000130 ***	0.000291 ***
Percent of adults with bachelor’s degree	0.150 ***	0.152 ***	0.155 ***
Percentage of population aged 65 and above	14.84 ***	14.38 ***	12.71 ***
Rates of Female	−10.99 ***	−11.73 ***	−7.695 **
Number of days of COVID-19 vaccine availability	−0.118 ***	−0.121 ***	−0.135 ***
1 February 2021	1.352 ***	1.352 ***	1.352 ***
1 March 2021	6.718 ***	6.718 ***	6.718 ***
1 April 2021	15.37 ***	15.38 ***	15.40 ***
1 May 2021	28.47 ***	28.54 ***	28.91 ***
2 June 2021	37.59 ***	37.75 ***	38.54 ***
2 July 2021	44.25 ***	44.49 ***	45.69 ***
Racial minorities *Unemployment rate		0.737 ***	
Racial minorities * Per capita income			−0.000915 ***
Constant	−6.185 ***	−4.843 ***	−11.31 ***
Observations	19,999	19,999	19,999
R-squared	0.758	0.758	0.761

*** *p* < 0.001, ** *p* < 0.01, * *p* < 0.05.

## Data Availability

Publicly available datasets were analyzed in this study. This data can be found here: https://www.census.gov/programs-surveys/ces/data/restricted-use-data/demographic-data.html (accessed on 30 May 2021) and here https://data.cdc.gov/Vaccinations/COVID-19-Vaccinations-in-the-United-States-County/8xkx-amqh (accessed on 30 May 2021).
